# Supplementary material for: Putting It All Together: The Roles of Ribosomal Proteins in Nucleolar Stages of 60S Ribosomal Assembly in the Yeast Saccharomyces cerevisiae
Source: Biomolecules. 2024 Aug 9;14(8):975. doi: 10.3390/biom14080975 (PMC11353139; doi:10.3390/biom14080975)
Supplement: Supplementary file 1 [file biomolecules-14-00975-s001.zip › Supplemental/Supplemental-Figure-Legends-UPDATE-aug8.docx]

**Figure S1.** (A) Phylogenetically conserved secondary structure of LSU rRNA. The 5S rRNA, 5.8S rRNA, and six domains of 25S rRNA are color coded. The six root helices are boxed and highlighted [1,2] ; (B) The tertiary structure of rRNA in the LSU, color coded as in A; (C) The tertiary structure of the six LSU root helices, color coded as in A; (D) The RPs in the mature LSU (color coded as in A). The RPs colored in white are not discussed in this review. Abbreviations: proximal stem (PS), central protuberance (CP) (PDB: 4V88) [47].

**Figure S2.** (A) Cartoon depicting transcription of rRNA and early stages of small and large subunit assembly. Co-transcriptional assembly of some RPs is indicated. In co-transcriptional assembly, endonucleolytic cleavage occurs in the nascent rRNA at site A_2_ to separate the pathway of assembly for the two subunits (pre-40S, pre-60S). In post-transcriptional assembly, cleavage occurs at the A_3_ site only after transcription has completed; (B) Pathway of endo- and exonucleolytic processing of pre-rRNA. The co- and post-transcriptional pathways are shown; (C) Pathway of RP assembly into the LSU. The order is based upon when each RP is first visualized by cryo-EM. RPs in each neighborhood are color coded as in Figure S1. RPs shown in grey are not discussed in this review. Note that L41 is visualized in a few fungal LSUs, but otherwise is only observed in the SSU of archaea and eukaryotes, and thus is designated as eS32 in these organisms (M. Leibundgut and N. Ban, personal communication). PDB files for each structure are shown. Abbreviations: proximal stem (PS), central protuberance (CP).

**Figure S3.** Assembly pathway of the LSU showing consecutive nuclear intermediates discussed in this review. This schematic details the association of RPs and the entry and exit of AFs as visualized by cryo-EM. Note that some proteins may be present at other points in assembly but may not be resolved in a structural intermediate. PDB files are indicated for each structure. RPs are color coded according to their neighborhood (Figure S1), whereas all AFs are colored in cyan (first row). Consecutive assembly of RPs (second row). Entry and exit of AFs (third row). Visualization of rRNA tertiary structure as it undergoes stabilization (fourth row). Secondary structure of resolved rRNA. Segments not resolved are shown as transparent (fifth row). Adapted from [18].
