# Supplementary figures and images for: Putting It All Together: The Roles of Ribosomal Proteins in Nucleolar Stages of 60S Ribosomal Assembly in the Yeast Saccharomyces cerevisiae
Source: Biomolecules. 2024 Aug 9;14(8):975. doi: 10.3390/biom14080975 (PMC11353139; doi:10.3390/biom14080975)

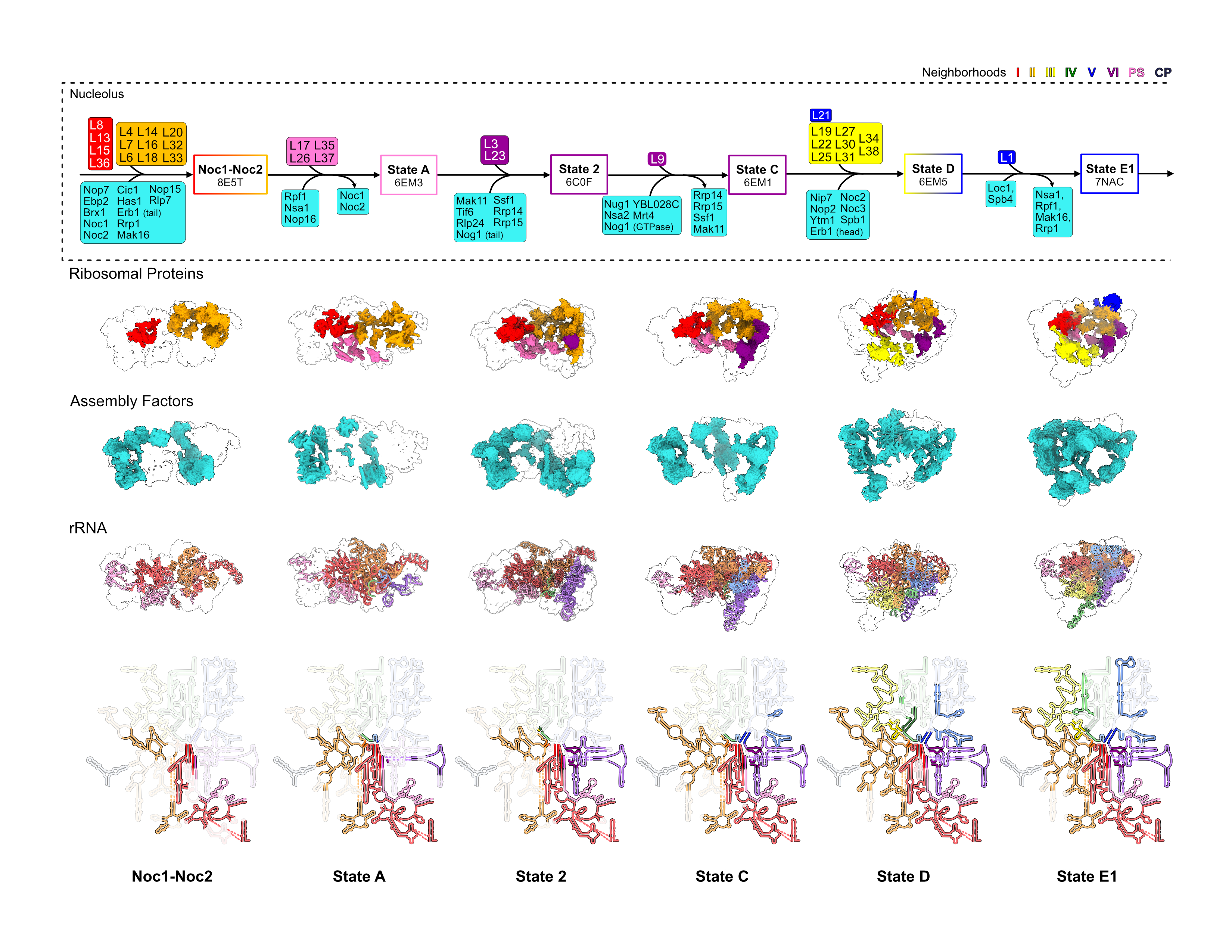

Supplement: Supplementary file 1 [file biomolecules-14-00975-s001.zip › Supplemental/FigureS3-pg1.jpg]

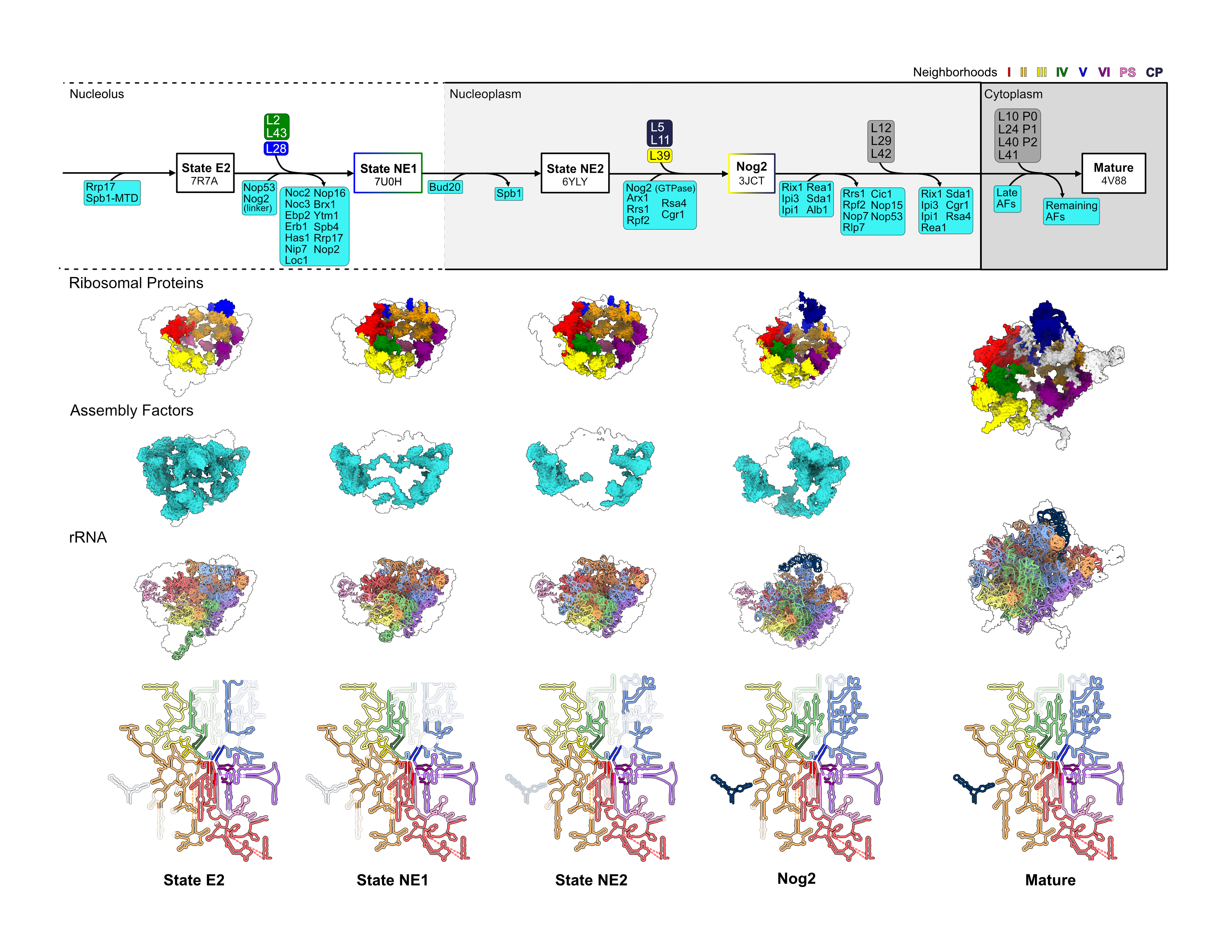

Supplement: Supplementary file 1 [file biomolecules-14-00975-s001.zip › Supplemental/FigureS3-pg2.jpg]

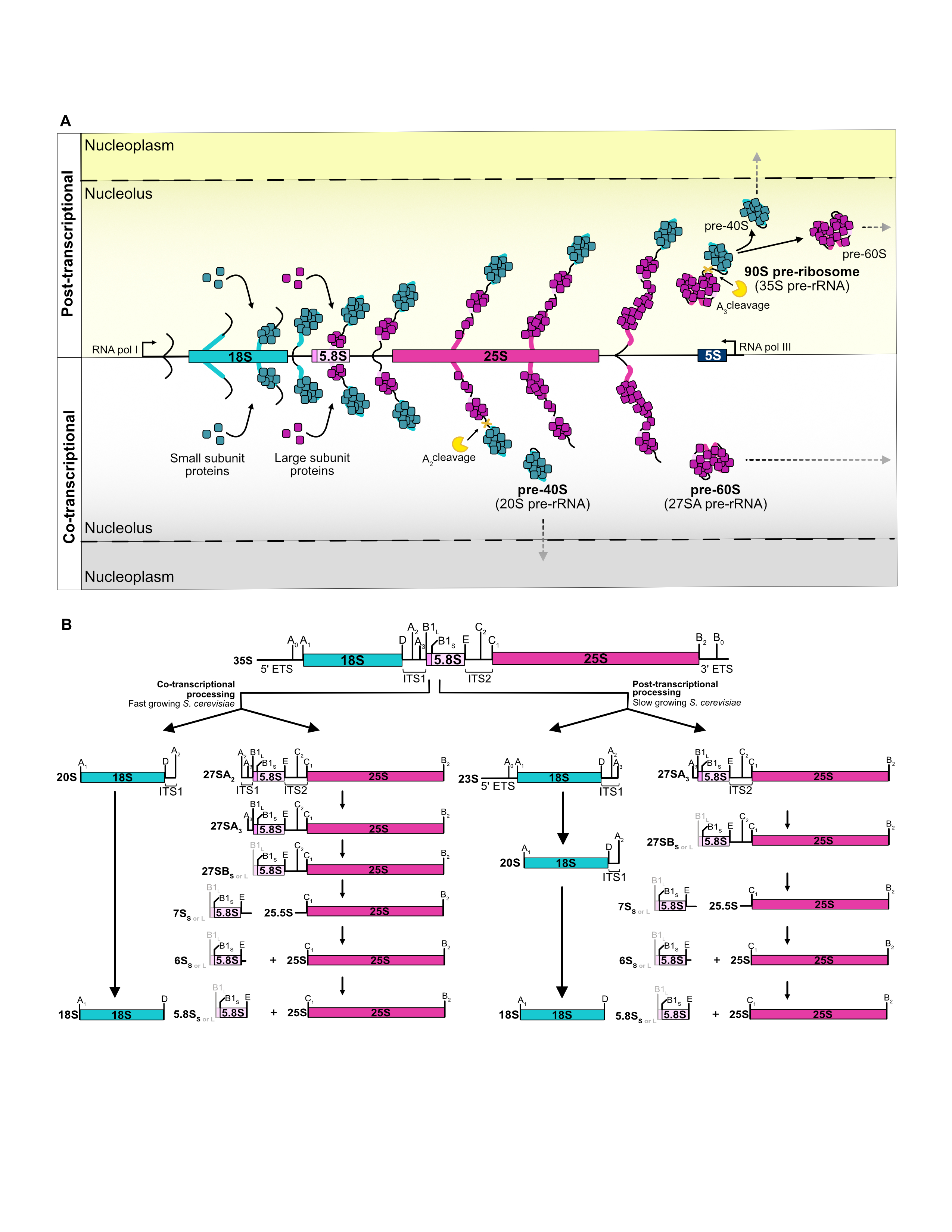

Supplement: Supplementary file 1 [file biomolecules-14-00975-s001.zip › Supplemental/FigureS2A-S2B.jpg]

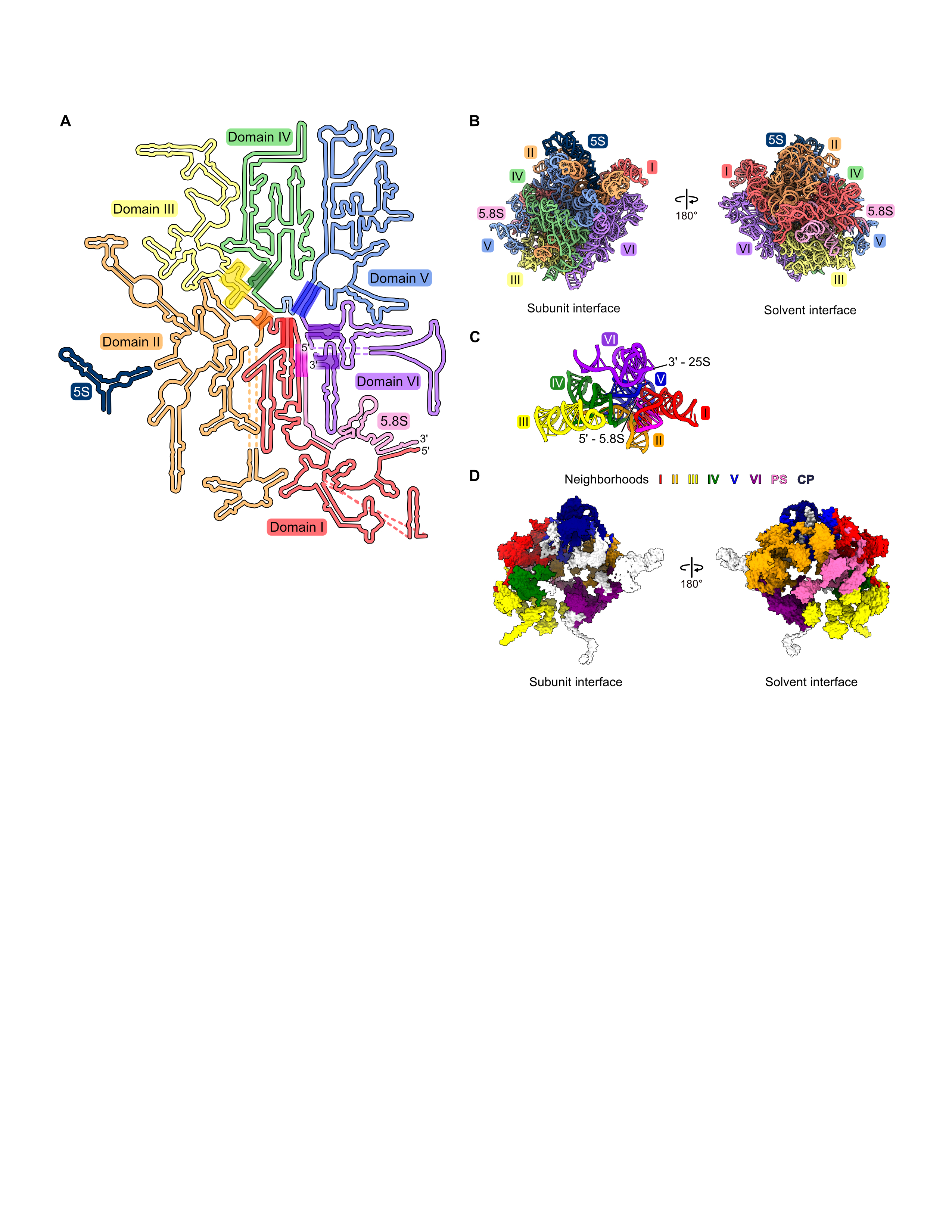

Supplement: Supplementary file 1 [file biomolecules-14-00975-s001.zip › Supplemental/FigureS1.jpg]

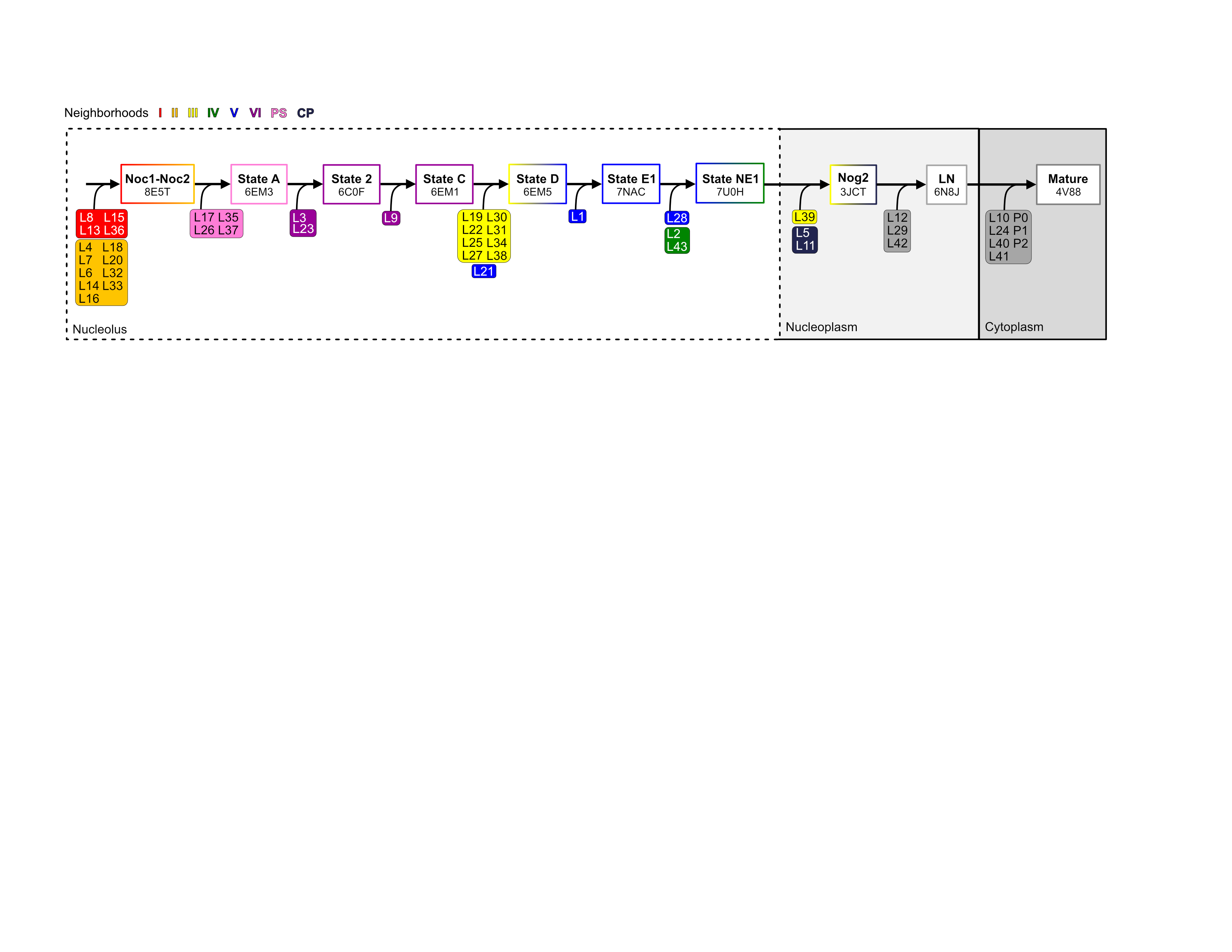

Supplement: Supplementary file 1 [file biomolecules-14-00975-s001.zip › Supplemental/FigureS2C.jpg]
